# Does mhGAP training of primary health care providers improve the identification of child- and adolescent mental, neurological or substance use disorders? Results from a randomized controlled trial in Uganda

**DOI:** 10.1017/gmh.2018.18

**Published:** 2018-09-10

**Authors:** A. Akol, F. Makumbi, J. N. Babirye, J. S. Nalugya, S. Nshemereirwe, I. M. S. Engebretsen

**Affiliations:** 1 Centre for International Health, University of Bergen, Bergen, Norway; 2 School of Public Health, Makerere University College of Health Sciences, Kampala, Uganda; 3 Department of Psychiatry, Mulago Hospital Kampala, Kampala, Uganda; 4 Butabika National Mental Referral Hospital, Kampala, Uganda

**Keywords:** Adolescent, Africa, child, mental health, mhGAP, primary care

## Abstract

**Background.:**

Integrating child and adolescent mental health (CAMH) into primary health care (PHC) using the WHO mental health gap action program (mhGAP) is recommended for closing a mental health treatment gap in low- and middle-income countries, but PHC providers have limited ability to detect CAMH disorders. We aimed to evaluate the effect of PHC provider mhGAP training on CAMH disorder identification in Eastern Uganda.

**Methods.:**

Thirty-six PHC clinics participated in a randomized controlled trial which compared the proportion of intervention (*n* = 18) to control (*n* = 18) clinics with a non-epilepsy CAMH diagnosis over 3 consecutive months following mhGAP-oriented CAMH training. Fisher's exact test and logistic regression based on intention to treat principles were applied. (clinicaltrials.gov registration NCT02552056).

**Results.:**

Nearly two thirds (63.8%, 23/36) of all clinics identified and recorded at least one non-epilepsy CAMH diagnosis from 40 692 clinic visits of patients aged 1–18 recorded over 4 months. The proportion of clinics with a non-epilepsy CAMH diagnosis prior to training was 27.7% (10/36, similar between study arms). Training did not significantly improve intervention clinics’ non-epilepsy CAMH diagnosis (13/18, 72.2%) relative to the control (7/18, 38.9%) arm, *p* = 0.092. The odds of identifying and recording a non-epilepsy CAMH diagnosis were 2.5 times higher in the intervention than control arms at the end of 3 months of follow-up [adj.OR 2.48; 95% CI (1.31–4.68); *p* = 0.005].

**Conclusion.:**

In this setting, mhGAP CAMH training of PHC providers increases PHC clinics’ identification and reporting of non-epilepsy CAMH cases but this increase did not reach statistical significance.

## Introduction

One in five children globally (Belfer, 2008) and one in seven in sub-Saharan Africa (Cortina *et al*. 2012) suffers a mental, neurological or substance use (MNS) disorder, but the coverage of child and adolescent mental health (CAMH) services in most low- and middle-income countries (LMIC) is poor (Patel *et al*. 2007, 2008). Global discussions in the last decade have recommended task-shifting to integrate mental health services into PHC as a means of improving CAMH service access (Eaton *et al*. 2011; Kieling *et al*. 2011; World Health Organization, 2003; 2008a; b; World Health Organization *et al*. 2008).

Task shifting is a strategy designed to improve the efficiency of available human resources for health through redistribution of selected roles from highly qualified to lower-trained personnel, freeing up the highly qualified staff to pursue more specialized roles (World Health Organization, 2007). Task-shifting has increasingly been applied to various healthcare systems over the last decade, with demonstrated dividends for population health and cost-saving (Seidman & Atun, 2017). In Uganda, task-shifting strategies in which tasks are typically transferred from medical doctors to nurses, or nurses to community health workers are conspicuous in HIV/AIDS care (Kalibala *et al*. 2016), maternal and child health (Nabudere *et al*. 2011), family planning (Janowitz *et al*. 2012) and have been explored for non-communicable disease management (Katende & Donnelly, 2016). Task-shifting has also been shown to improve reach and effectiveness of mental health services in low-resource settings with scarce human resources (Hoeft *et al*. 2018).

Available literature from Uganda suggests that task-shifting in mental health systems is a necessary, feasible and acceptable strategy (Mendenhall *et al*. 2014; Mugisha *et al*. 2017) which has been applied successfully in scaling up child mental health services in schools (Huang *et al*. 2015). However, the ability of PHC providers to detect and manage CAMH conditions is lacking (Patel *et al*. 2007). Consequently, mental health services are not integrated into PHC services in spite of several available opportunities (Ovuga *et al*. 2007; Kigozi & Ssebunnya, 2009; Lund *et al*. 2016) and the mental health needs of children and adolescents in Uganda largely go unmet (Ministry of Health Uganda, 2017).

Whereas the burden of CAMH disorders in Uganda has not been accurately estimated, mental health professionals agree that mental ill-health is an increasing problem, exacerbated by poor access to mental health services (Akol *et al*. 2015). Currently, mental health services are provided in a centralized framework at national and regional hospitals, contrary to WHO recommendations for mental health care in low-income countries to be provided at PHC clinics and in communities (World Health Organization, 2001, 2008*a*; Kigozi *et al*. 2010; Akol *et al*. 2015). Health system research aimed at increasing the availability of and access to mental health care for children and adolescents in settings like Uganda is an important public health priority (Patel *et al*. 2007; Tomlinson *et al*. 2009).

WHO's mental health gap action program (mhGAP) and its attendant intervention guide (IG) were developed through rigorous, evidence-based methodologies, to aid integration of selected, priority MNS disorders, including developmental and behavioral disorders in children and adolescents, into PHC services in LMIC. The IG provides algorithms for simplified clinical assessment, diagnosis, management, follow-up and referral were indicated as well as a trainer's guide to facilitate standardized implementation (World Health Organization, 2010). Based on evidence and extensive feedback from mhGAP-IG practitioners, the WHO in 2016 launched a second version of the mhGAP-IG with revised modules for Child and Adolescent Mental and Behavioural Disorders (World Health Organization, 2017). Several countries in sub-Saharan Africa are implementing mhGAP; a 2017 systematic review of studies reporting mhGAP implementation concluded that the program has been beneficial to mental health care in LMIC.

**Fig. 1.**
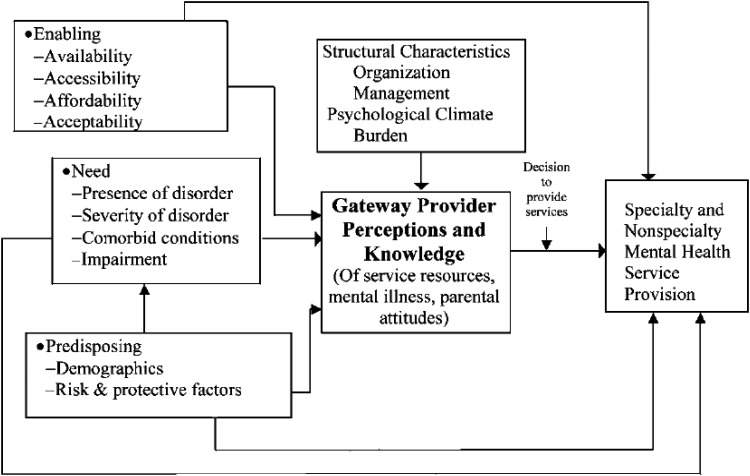
The Gateway Provider Model of youth access to mental health services, from Stiffman et al. (2004).

Stiffman and colleagues’ Gateway Provider Model (Fig. 1) addresses mental health service access and articulates the importance of decision-makers who influence health system trajectories into care. In the context of children and adolescents, these decision makers are called Gateway Providers, and their knowledge of and attitudes to CAMH influence access to care (Stiffman *et al*. 2004). In rural Uganda, PHC providers are an important category of gateway provider (Kigozi & Ssebunnya, 2009*a*) whose role in increasing access to CAMH services in Uganda is unknown. Further, scanty literature exists on the effectiveness of integrating CAMH into PHC through mhGAP-IG implementation in LMIC. This study aimed to evaluate the effect of a PHC provider mhGAP-IG-oriented CAMH training on identification of MNS disorders among children and adolescents in Eastern Uganda.

## Methods

### Study area

The study was conducted in Mbale and Sironko districts in Eastern Uganda, selected because both were mhGAP naïve; and Mbale was the site for previous CAMH work in the ‘SeeTheChild—Mental Child Health in Uganda’ (SeeTheChild) study (Center for International Health), which included Sironko district in its catchment area. We concluded that there was no mhGAP exposure in the districts prior to our study based on our knowledge of the area, and on confirmation with the district health authorities.

The two districts possess all levels of health service, including rural health posts and a psychiatric unit at the regional referral hospital in Mbale town. They are mostly rural, characterized by a steep mountainous terrain that presents a physical barrier to psychiatric and other health services. Pathways to mental health care typically include traditional healers (Abbo, 2011; Akol *et al*. 2015), and psychiatric referral facilities where they exist (Kisa *et al*. 2016).

The study included Level-3 health clinics (HC III) because they are the lowest level at which comprehensive PHC services are provided. In addition, the presence of at least four staff at the HC III enabled two staff members to attend CAMH training without disabling service delivery. In 2014, Mbale district had 25 public HC-III compared with 12 in Sironko. At these HC III, PHC services are provided by clinical officers, nurses and midwives. Clinical officers have 3 years of pre-service training compared with nurses and midwives who hold a certificate following 1–2 years of pre-service training. Exposure to MNS disorders during pre-service training varies for these cadres; clinical officers’ training typically has more content on MNS disorders than a nurse or midwife training (Collins *et al*. 2015).

### Study design

This was a randomized controlled study that compared the clinic-level outcomes of CAMH training on CAMH case identification and recording compared with non-CAMH trained sites. A list of all eligible clinics in Mbale and Sironko districts was obtained from the district health offices and randomly assigned to intervention and control by an independent collaborator who was separate from the research team, using computer-generated random numbers. In randomizing, first, the clinic list was sorted alphabetically to eliminate clinic sorting by district before a random sequence number was generated using the command ‘randomize’ in stata v.12 (StataCorp Texas, USA 2011). This method resulted in 18 clinics being allocated each to the intervention and control arms.

### Inclusion and exclusion criteria

Clinics were included if they were HC IIIs owned and managed under the government health system in the two districts; and excluded if they had a psychiatric nurse or clinical officer on staff or had been exposed to the mhGAP-IG prior to the study. None of the clinics met the exclusion criteria.

### Sample size calculation

The sample size determination was done using the formula by Fleiss for sample size in cohorts and trials (Fleiss *et al*. 2013). The following assumptions were made: estimated CAMH diagnosis rate of −0.5% (based on district records), a 50% detection rate in exposed clinics, a power of 90%, the type-I rate of 5% with a two-sided confidence interval. Applying these assumptions gives us an estimated number of 18 clinics required in each arm.

### Procedures for intervention

#### Control arm

Treatment as usual, characterized by routine PHC services to children and adolescents

### Intervention package

1. Training PHC workers (midwives, nurses and/or clinical officers) on how to screen and refer for priority CAMH conditions, based on the WHO mhGAP-IG v1.
2. Provision of reference training materials (handouts from the training slides) on patient screening using the mhGAP-IG v1.

*Training*: Two PHC workers from each intervention clinics were trained for 5 days to screen children and adolescents for CAMH conditions, based on the mhGAP-IG curriculum version 1.0 (World Health Organization, 2010). Most of the PHC workers were nurses or midwives and just over 40% had been in service for less than 5 years. The aim of the training was to equip the PHC workers with knowledge and skills in assessment, identification and treatment or referral of children and adolescents with mental health problems so as to contribute to early referral of children into care. An account of the PHC workers, training and outcomes is published separately (Akol *et al*. 2017).

### Blinding

PHC workers could not be blinded to the intervention. However, we did not independently check patients’ awareness of the training. Data entry personnel and the two research assistants (RAs) who were responsible for capturing clinic records were blinded to clinic allocation.

### Data collection

The principal investigator and two RAs (clinicians resident in the districts, familiar with the district health system) captured service delivery records containing information on patient age, sex, residence and diagnosis from clinic registers, using electronic tablets. Data were collected for the month preceding the intervention (baseline) and for each of 3 months subsequent to the intervention (follow-up months 1, 2 and 3). For each patient, clinic registers include a provision for a first and second diagnosis to enable recording of co-morbidities. Both first and second diagnoses were captured.

### Data processing

Records were then entered into Epidata V3.1 (The EpiData Association, Odense, Denmark) by a team of trained and experienced data entrants. Epidata files were exported to STATA v13 (statacorpIC) for cleaning and analyses.

Explanatory and outcome variables were treated as follows: first, only patients aged 1–18 years were selected, and patient age was re-categorized into 1–4, 5–9,10–14 and 15–18 age groups. Second, all diagnoses were reclassified and re-coded into WHO's International Statistical Classification of Diseases and Related Health Problems, version 10 (ICD-10) categories (World Health Organization, 2004). In doing this, all possible diagnoses in the clinic register fitting under an ICD-10 category were replaced with the ICD-10 category name using appropriate commands in stata. Thus, the ICD-10 category ‘Mental and Behavioral’ replaced all mental health diagnoses. Mental and Behavioral disorders were further classified according to the fifth edition of the Diagnostic and Statistical Manual of Mental disorders, (DSM V) (American Psychiatric Association, 2013) and the frequencies analyzed by study arm and month of study.

### Outcome measures

The primary outcome was the proportion of clinics in the intervention relative to the control arm that diagnosed and recorded at least one non-epilepsy CAMH case over the 3-month follow-up period while the secondary outcome was the likelihood of patients receiving a non-epilepsy CAMH diagnosis in the intervention compared with the control arms. Epilepsy was excluded based on available information showing that epilepsy is already being diagnosed and managed in PHC settings (Kigozi *et al*. 2010; Lund *et al*. 2016).

### Statistical methods

We used Intention to Treat principles. The unit of analysis was a health facility, and the primary outcome was a facility reporting at least a diagnosed non-epilepsy CAMH case, coded as 1:yes or else 0:no if no such case was reported. We compared the primary out between study arms using Fisher's exact test (Fisher, 1922). The choice of analysis approach was decided based on the number of expected observations in the table cells that were five or less in either or both study arms. Statistical significance was determined at *p* < 0.05.

The secondary outcome was ‘patients diagnosed with non-epilepsy CAMH’. All patients diagnosed with non-epilepsy CAMH were coded as 1 = yes or 0 = no if no such diagnosis was made. The odds of a non-epilepsy CAMH diagnosis were compared between intervention and control arm to obtain the odds ratio (OR) as the measure of association. The ORs were obtained via a logistic regression model with corresponding 95% confidence intervals (CI). In the multivariable logistic regression model, factors such as age and sex were included. All models accounted for clustering of observations at the clinic level to obtain robust standard errors of the estimates. Stata version 14 was used for all the analyses.

### Role of the funding sources

The study sponsors had no role in study design or management; data collection or handling; interpretation of findings or writing of the paper. AA, IMSE and JNB had full access to all study data and are responsible for the decision to submit for publication.

## Results

**Fig. 2.**
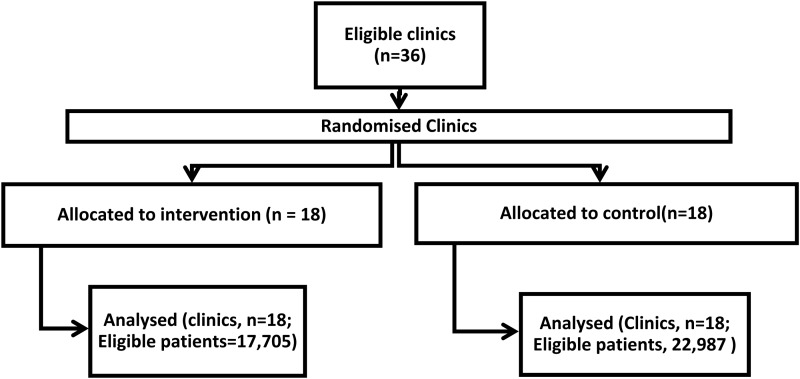
CONSORT flow diagram.

Figure 2 shows the trial profile, including all clinics and patients aged 1–18 whose data was analyzed.

Forty-two percent of clinics in Mbale (*n* = 11) and 46.2% in Sironko (*n* = 7) were allocated to the intervention arm. Clinics were equally distributed by the district in the intervention and control arms, but the number of patient visits was significantly different (*p* < 0.001) in intervention and control clinics (Table 1).

**Table 1.** Characteristics of clinics by study arm

Total	Intervention N (%)	Control N (%)	*p* value
	18 (100)	18 (100)	
District			0.480
Mbale	11(61.1)	13 (72.2)	
Sironko	7 (38.9)	5 (27.8)	
Under 19 patient visits			
Total	17705 (100)	22987 (100)	<0.001
Baseline	4310 (24.3)	5003 (21.8)	
Follow-up month 1	4210 (23.8)	5752 (25.0)	
Follow-up month 2	4773 (27.0)	7084 (30.8)	
Follow-up month 3	4412 (24.9)	5148 (22.4)	

Participants were equally distributed across study arms for age group and sex (Table 2). Clinics in the intervention arm received 44.5% of all patients seen across the 4 months. Equal proportions of patients were seen at intervention and control clinics in all months, except in November, when increased numbers of patients were recorded in both intervention and non-intervention clinics. Sixty percent of patients in both arms were female and the mean age of patients across study arms was 9 years.

**Table 2.** Background characteristics of patients 1–18 years by study arm

	Baseline month		Study month 1		Study month 2		Study month 3	
	Intervention *N* = 4310 *N* (%)	Control *N* = 5003 *N* (%)	Intervention *N* = 4210 *N* (%)	Control *N* = 5752 *N* (%)	Intervention *N* = 4773 *N* (%)	Control *N* = 7084 *N* (%)	Intervention *N* = 4412 *N* (%)	Control *N* = 5148 *N* (%)
Age group								
1–4	1122 (26.0)	1391 (27.8)	1064 (25.3)	1508 (26.2)	1358 (28.5)	2109 (29.8)	1159 (26.3)	1523 (29.6)
5–9	1014 (23.5)	1202 (24.1)	984 (23.4)	1513 (26.3)	1257 (26.3)	1925 (27.2)	1285 (29.1)	1393 (27.1)
10–14	1249 (29.0)	1323 (26.4)	1244 (29.5)	1712 (29.8)	1288 (27.0)	1745 (24.6)	1061 (24.0)	1160 (22.5)
15–18	925 (21.5)	1087 (21.7)	918 (21.8)	1019 (17.7)	870 (18.2)	1305 (18.4)	907 (20.6)	1072 (20.8)
Sex								
Male	1662 (38.6)	1944 (38.9)	1690 (40.1)	2282 (39.7)	1870 (39.2)	2746 (38.8)	1647 (37.3)	1932 (37.5)
Female	2524 (58.6)	2958 (59.1)	2487 (59.1)	3397 (59.0)	2796 (58.6)	4254 (60.0)	2729 (61.9)	3184 (61.9)
Missing	124 (2.9)	101 (2.0)	33 (0.8)	73 (1.3)	107 (2.2)	84 (1.2)	36 (0.8)	32 (0.6)
District								
Mbale	3055 (70.9)	3818 (76.3)	3043 (72.3)	3990 (69.4)	3135 (65.7)	5362 (75.7)	2939 (66.6)	3385 (65.8)
Sironko	1255 (29.1)	1185 (23.7)	1167 (27.7)	1762 (30.6)	1638 (34.3)	1722 (24.3)	1473 (33.4)	1763 (34.2)

### Disease profile

Respiratory conditions were the most prevalent across the follow-up months (40.5%); followed by infectious and parasitic infections (39.6%). Mental and behavioral conditions represented 0.1% (*n* = 64) of all diagnoses recorded. Except for mental and behavioral conditions, all other disease categories were equally distributed between the two arms (data not shown).

### Types of CAMH cases diagnosed

Twenty-three clinics overall (63.8%) recorded a CAMH diagnosis; 14 (77.8%) of these were in the intervention arm. Over the 3 months’ follow-up period the number of CAMH-diagnoses at intervention clinics ranged from 0 to 7, with half (*n* = 18) of the diagnoses in the intervention arm being recorded by three clinics. On the other hand, in control clinics, CAMH diagnoses ranged from 0 to 5 in number with one clinic providing 36% of diagnosed patients (data not shown).

Table 3 presents the CAMH diagnoses. The most prevalent disorders were somatic symptom disorders (31.3%, *n* = 20), accounting for two-thirds of all CAMH diagnoses recorded in the baseline month, 44.4% (8/18) of all CAMH disorders identified in the control and 26% (12/46) in the intervention arms.

**Table 3.** CAMH profile by month and study arm

	Baseline Month (*n*)		Study month 1 (*n*)		Study month 2 (*n*)		Study month 3 (*n*)		Totals (*n*)^a^	
	Int	Control	Int	Control	Int	Control	Int	Control	Int	Control
Anxiety disorders	0	1	2	0	5	2	4	2	11	5
Depressive disorders	0	0	1	0	5	0	0	1	6	1
Elimination disorders	0	0	0	0	2	0	4	0	6	0
Intellectual disability	0	0	3	0	0	0	1	0	4	0
Schizophrenic spectrum	1	1	0	0	0	1	2	0	3	2
Somatic symptom	6	2	5	3	0	0	1	3	12	8
Trauma and stressor	1	0	2	1	0	1	0	0	4	2
Totals	8	4	13	4	12	4	12	6	46	18

int, intervention arm.

a 64 diagnoses were recorded from 63 unique patients.

### Primary outcome

A total of 20 out of 36 clinics made and recorded at least one CAMH diagnosis over the 3-month follow-up period. The proportion of clinics with a CAMH diagnosis tended to be higher in the intervention (13/18, 72.2%) relative to the control (7/18, 38.9%) arm but this difference was not statistically significant (*p* = 0.092) (Table 4).

**Table 4.** Percent of clinics with CAMH diagnosis by study arm

Study Month	Percent of clinics with CAMH diagnosis		Fishers Exact test
	Intervention	Control	
Baseline month	33.3	22.2	0.711
Follow-up month 1	38.9	11.1	0.121
Follow-up month 2	33.3	22.2	0.711
Follow-up month 3	44.4	22.2	0.289
Follow-up months 1–3	72.2	38.9	0.092

### Secondary outcome

Over the 3-month follow-up period, the adjusted odds of a patient being diagnosed with a CAMH case were 3.4 times higher in the intervention relative to the control arm, and this effect was statistically significant [aOR = 3.38; 95% CI (1.34–8.52), *p* = 0.010]. The effect of the intervention on patient diagnosis was highest in the first follow-up month [aOR 4.16; 95% CI (0.73–23.72), *p* > 0.05] and decreased sequentially for the second [aOR 3.82, 95% CI (1.16–12.60), *p* = 0.027)]and third [aOR 2.56, 95% CI (0.80–8.21), *p* > 0.05] follow-up months. The odds of a CAMH diagnosis in the third follow-up month were the same as the odds of a CAMH diagnosis in the baseline month (Table 5).

**Table 5.** Logistic regression of a CAMH diagnosis between study arms (controlling for age and sex, and accounting for clustering effects at clinic level)

	OR (95%CI)	aOR (95% CI)^a^
Baseline month	2.57 (0.78–8.55)	2.57 (0.86–7.69)
Follow-up month 1	4.16 (1.36–12.78)**	4.16 (0.73–23.72)
Follow-up month 2	3.82 (1.23–11.86)**	3.82 (1.16–12.60)**
Follow-up month 3	2.56 (0.96–6.81)	2.56 (0.80–8.21)
Follow-up months 1–3	3.38 (1.83–6.26)**	3.38 (1.34–8.52)**

a Accounting for clustering effects at the clinic level.

** *p* < 0.05.

At baseline, the odds of diagnosing a patient with a non-epilepsy CAMH condition were higher in the intervention than in the control arm, but this was not statistically significant; this finding may suggest some level of residual imbalance in the assigned study arms. Therefore, the final model was adjusted for baseline identification of CAMH at the facility level. This model confirmed that clinics in the intervention arm had 3.4 times higher odds of diagnosing CAMH in the intervention relative to control arm clinics, [aOR 3.38; 95% CI (1.34–8.52); *p* = 0.010].

## Discussion

This study examined the effect of CAMH training on non-epilepsy CAMH case identification among PHC providers in Eastern Uganda. We found no significant difference in the proportion of intervention and control clinics diagnosing non-epilepsy CAMH. However, the odds of a patient being diagnosed with non-epilepsy CAMH were significantly higher in the intervention than control clinics.

To our knowledge, this study is one of the first on CAMH integration into PHC in Uganda, and one of the first to report clinic-level impacts of PHC worker CAMH training. Available studies on mental health training for PHC providers are oriented to adult-psychiatry and most report provider-level effects (Liu *et al*. 2016). Our study found that mhGAP CAMH training of PHC workers did not significantly affect clinic-level non-epilepsy CAMH diagnoses. Similarly, a cluster randomized trial in Kenya revealed that non-specialist health worker (NSHW) mental health training improved patient outcomes but did not enhance the detection of mental health problems in primary health care (Jenkins *et al*. 2013*b*).

We attribute the lack of impact to three factors: first, the CAMH diagnoses made over the 3-month follow-up period (51 in both intervention and control arms) were likely too few to generate a visible effect. The scantiness of diagnoses might be a result of a low prevalence of non-epilepsy CAMH illness in this community, estimates of which are not available. Another likely reason is a poor help-seeking behavior to the public health system for non-epileptic CAMH conditions. However, we were unable to find evidence of this in the CAMH literature in settings similar to Uganda's. Third, half the diagnoses in the intervention clinics came from three clinics; and over a third in the control clinics were provided by one facility. This suggests unique motivational factors affecting providers in those facilities, skewing the diagnoses towards a few clinics. Such system-level factors like patient load, health worker numbers and their skills-mix, as well as clinic management have been identified as factors that influence health system performance (Murray & Frenk, 2000; Rowe *et al*. 2005; Jerene *et al*. 2017). However, we did not investigate these factors in our study.

Nevertheless, we found significantly higher CAMH diagnoses in intervention than in control clinics. Previous studies evaluating task-shifting for mental health in similar contexts have also reported positive results. Gureje *et al*. found analogous results in their 2015 study in Nigeria (Gureje *et al*. 2015) and a 2013 Cochrane review found that the use of NSHW resulted in positive treatment outcomes compared with usual services for depression, anxiety, post-traumatic stress and alcohol use disorders in seven LMIC (Van Ginneken *et al*. 2013). This review was oriented to adult psychiatry and like our study, focused on NSHW training, though it assessed the utility of NSHW for improved treatment outcomes, rather than for enhanced detection of mental health conditions.

Except for the second follow-up month, we did not find any beneficial effect when data were examined on a month-on-month basis. Rather, the benefit was detectable after the 3-month follow-up period. The most plausible explanation for this is that the incidence and prevalence of CAMH cases in the two districts were too low to result in a perceptible difference at a single month, particularly since our intervention did not include community mobilization to encourage CAMH clinic attendance. Thus, over the 3-month follow-up period, only 64 CAMH cases were identified among 63 patients, corresponding to a clinic-prevalence rate of 0.1%. A study in neighboring Kenya was able to enroll 166 CAMH patients over an equivalent 12-week period (Kamau *et al*. 2017) but this study was conducted at a tertiary hospital in an urban setting. A possible explanation for the significant result in the second follow-up month is the comparatively higher under-19 patient visits in that month compared with the other follow-up months (51.6%, *n* = 11867).

We found that the effect of the intervention on non-epilepsy CAMH was strongest in the first follow-up month, reducing progressively until the third follow-up month when the effect size was equivalent to that in the baseline month. This finding strongly suggests that the knowledge and skills acquired during training diminish over time, requiring regular ‘refresher’ trainings. This suggestion is backed by similar findings elsewhere with PHC providers in sub-Saharan Africa (Gureje *et al*. 2015; Tilahun *et al*. 2017). In addition to declining health worker knowledge and skills, other health system weaknesses may account for a reduced effect on diagnoses over time. Jenkins *et al*. (2013*a*, *b*) report that inadequate information systems, district-level supervision and medicines supply may frustrate mental health integration into PHC (Jenkins *et al*. 2013*a*). These factors are present in Uganda's health system and may have played a role in declining performance (Kigozi *et al*. 2010; Akol *et al*. 2015).

### CAMH case profile

We found a low CAMH morbidity rate with a high prevalence of somatic-symptom and anxiety disorders. The CAMH disorder profile we found is similar to that seen in a recent study in urban Kenya which found that alcohol and substance use disorders were the most prevalent, followed by depression and anxiety-related disorders (Kamau *et al*. 2017). Alcohol use disorders were not identified in our study but somatic symptom disorders were commonly diagnosed in the baseline month and by clinics in the control arm, suggesting that the ability of PHC workers to identify them is independent of CAMH training. This proposition is supported by recent opinions suggesting that the DSM-V somatic symptom diagnostic criteria are so inclusive that they increase the risk of healthy patients being classified as mentally ill (Frances, 2013). This over-diagnosis is a possibility we can neither rule out nor confirm in our study since we did not validate the PHC providers’ diagnoses.

### Strengths and limitations

The following limitations should be considered in the interpretation of our study findings. First, we recognize the reliance on routinely collected health information from clinic-based registers, which have known limitations with completeness and correctness of entries (World Health Organization, 2011). We excluded several records from the study for incomplete or incorrect reporting of either age or diagnosis or both. Thus, though we cannot quantify the excluded records, we believe that the prevalence of CAMH disorders may be under-estimated in this study.

Second, our estimated sample size was based on a 0–1% detection rate in the unexposed, based on CAMH-diagnosis records in the District Health Information System (DHIS). An estimate closer to the rate we found in the un-exposed clinics would have yielded a larger sample size. Thus, using estimates from DHIS led to a small sample size, effectively under-powering the study. This might explain the lack of statistical significance in the primary outcome despite the higher percent (72.2%) of the intervention compared with control (38.9%) clinics with a CAMH diagnosis. Third, during data collection, we encountered several instances in which trained staff were absent from the clinics for extended periods. Such absenteeism was foreseen; hence the training of two PHC providers per clinic. Thus, much as all intervention clinics retained at least one trained staff for the duration of the study, we cannot exclude the likelihood that not all patients received CAMH screening, with consequent under-detection of CAMH cases.

Fourth, we did not consider clinic characteristics such as staff numbers per clinic and health worker cadre and work experience in analysis. Lack of data on these potential confounders may limit our ability to make more clear conclusions about the effect of the intervention on diagnosing of non- epilepsy CAMH. Finally, although the intervention and control arms shared several similarities, they were dissimilar in the number of patient visits during the follow-up period. This bias was addressed by adjusting for the clinic variable in the multivariable analyses. On the other hand, we note some strengths of this study. The robust study design we applied enables us to draw conclusions about the utility of PHC provider CAMH training on the studied clinic-level CAMH outcomes. Additionally, we addressed the potentially confounding effect of clinics’ baseline ability to identify CAMH cases by adjusting for this factor in the multivariable logistic regression.

## Conclusions

In this setting, mhGAP CAMH training of PHC providers increases PHC clinics’ identification and reporting of non-epilepsy CAMH cases but this increase is not statistically significant. However, training significantly improves the likelihood of a non-epilepsy CAMH diagnosis.

This study utilized a random selection of all public HC III in two districts which are similar in health system characteristics to other rural Ugandan districts; therefore, the results from this study are generalizable to other HC III in Uganda and other similarl rural settings in sub-Saharan Africa. Our findings suggest that much as CAMH training of PHC providers in Uganda would not result in improved clinic-level competence to diagnose CAMH conditions, it would lead to increased identification of CAMH disorders. Further task-sharing studies integrating CAMH into a larger sample of PHC clinics are suggested, including a community mobilization component in the intervention to improve CAMH clinic attendance.
